# Community-led monitoring of TB services in the Kyrgyz Republic

**DOI:** 10.5588/ijtldopen.25.0691

**Published:** 2026-07-13

**Authors:** B. Myrzaliev, A. Palastrov, L. Han, M. Ahmatov, E. Igolnikova, A. Kadyrov

**Affiliations:** 1 KNCV KG Office in the Kyrgyz Republic, Bishkek, Kyrgyz Republic;; 2 TB People Kyrgyz Republic, Bishkek, Kyrgyz Republic;; 3 National Center of Phthisiology, Ministry of Health, Bishkek, Kyrgyz Republic.

**Keywords:** tuberculosis, community engagement, patient-centred care, surveillance, service delivery, Kyrgyzstan

## Abstract

**BACKGROUND:**

TB remains a major public health challenge in the Kyrgyz Republic due to continued transmission and drug resistance. Despite an overall decline in incidence, gaps persist in case notification, procurement processes, and patient-centred care.

**METHODS:**

This study reports on the first community-led monitoring (CLM) initiative for TB services, implemented by TB People Kyrgyzstan in partnership with KNCV KG from 2019 to 2024. Programmatic data were combined with structured interviews of people with TB, community monitors, and health workers to identify barriers to diagnosis, treatment, and access to medicines.

**RESULTS:**

Estimated TB incidence declined over the study period, yet under-notification remained substantial, with 56.5% of cases not notified in 2024. Procurement was generally stable; however, regulatory and customs delays limited access to new medicines. Patients reported stigma, transport costs, and limited psychosocial support as key barriers, while health workers highlighted shortages of trained staff in rural areas.

**CONCLUSION:**

CLM complemented national surveillance by amplifying community perspectives and identifying actionable priorities. Expanding access to rapid diagnostics, strengthening procurement coordination, investing in the TB workforce, and sustaining community engagement are essential to accelerate progress towards End TB targets. This approach supports equity, improvement, and resilience.

TB remains a major public health challenge in the Kyrgyz Republic, with a persistent burden of drug-resistant disease and ongoing gaps in case detection and service delivery.^1–3^ Despite progress in expanding access to diagnostics and treatment, a substantial proportion of people with TB are not detected or are diagnosed late, contributing to ongoing transmission and poor outcomes. Strengthening patient-centred approaches and improving accountability within TB services are therefore critical priorities for the national response.^4^ Community-led monitoring (CLM) has emerged as an approach to systematically capture patient and community perspectives on health service delivery, providing complementary insights to routine surveillance systems and supporting more responsive and people-centred services. In the context of TB, CLM has been increasingly recognised as a tool to identify gaps in access, quality of care, and patient support, and to strengthen engagement between communities and health authorities.^5^

In the Kyrgyz Republic, TB services are delivered through a network of specialised facilities and primary health care providers. While national indicators show an overall decline in disease burden, challenges remain in timely diagnosis, case notification, access to new medicines, and continuity of care, particularly in rural and underserved areas. These challenges underscore the need for approaches that go beyond routine reporting and incorporate patient and community voices.^6^

This study presents findings from the first CLM initiative on TB services in the Kyrgyz Republic, assessing how policies and services are experienced by people with TB and frontline health workers and generating evidence to inform programmatic improvements within the National TB Program.

## METHODS

This study used a mixed-methods, observational design within the framework of CLM to identify service delivery gaps in TB care in the Kyrgyz Republic during 2019–2024 (Table 1). Quantitative programmatic indicators and qualitative accounts from people with TB and communities were systematically collected and analysed.

**Table 1.** Detection gap between estimated TB incidence and notified cases in the Kyrgyz Republic, 2019–2024, per 100,000 population (WHO country profile).

Year	Estimated TB incidence per 100,000 population	New and relapse TB cases notified per 100,000 population	Detection gap (absolute)	Detection gap (%)
2019	136	94.5	41.5	30.5
2020	131.2	63.6	67.6	51.5
2021	131.8	67.4	64.4	48.9
2022	132.8	65.7	67.1	50.5
2023	131.1	59.1	72.0	54.9
2024	127.6	55.5	72.1	56.5

Detection gap = estimated incidence − notified cases; detection gap (%) = (detection gap/estimated incidence) × 100.

The detection gap remained substantial throughout 2019–2024, ranging from 30.5% to 56.5%, indicating that up to more than half of estimated TB cases were not captured by the national notification system in recent years.

### Participants

Interviews were conducted with people with TB, community monitors, and health care workers involved in TB service delivery. Respondents were selected purposively to reflect geographic diversity and experience with TB services. Characteristics of respondents are summarised in Table 2.

**Table 2.** Characteristics of respondents.

Respondent group	Characteristics	n
People with TB	Male 7, female 5; urban 6, rural 6; drug-sensitive TB 8, drug-resistant TB 4; diagnosed 2019–2021: 5, diagnosed 2022–2024: 7	12
Health care workers	Male 3, female 5; TB doctors 4, nurses 2; programme staff 2; urban 5, rural 3; >10 years’ experience 4, ≤10 years’ experience 4	8
Community monitors	Male 1, female 2; urban 2, rural 1	3

### Community monitors

Data were collected by trained community monitors recruited from civil society organisations and TB-affected communities. Monitors received standardised training on CLM tools, ethical procedures, and data quality assurance.

### Implementation of community-led monitoring

CLM was implemented through structured interviews with people with TB and, where relevant, health care workers. Using standardised forms, monitors documented barriers across key stages of TB care, including access to diagnostics, medicines, and patient support, and shared consolidated findings with implementing partners and the National TB Program.

### Data sources

Primary CLM data were triangulated with secondary sources, including the WHO Global TB Report, National TB Program annual reports, and official statistics from the National Statistical Committee of the Kyrgyz Republic.

### Ethical statement

The study did not involve clinical interventions. All data were anonymised, informed consent was obtained, and principles of confidentiality and voluntary participation were upheld.

## RESULTS

The findings are presented below in four subsections: epidemiology, procurement and drug registration, case notification and access to care, and patient and community perspectives.

### Epidemiology

The long-term trend in TB in the Kyrgyz Republic indicates an overall decline compared with 2010, when the estimated incidence rate was 191.9 per 100,000 population and the notification rate for new and relapse TB was 102.9 per 100,000 population. This reflects strengthening of national TB services and improvements in diagnostic capacity; however, drug-resistant TB remains a major public health concern. During 2019–2024, fluctuations were observed within the overall downward trend. WHO estimates indicate a decline in TB incidence from approximately 136 to 127.6 per 100,000 population, while national notification rates remained consistently lower, reflecting persistent gaps in case detection and reporting. For example, in 2021, WHO estimated 131.8 incident cases per 100,000 population, whereas only 67.4 per 100,000 were notified through the national surveillance system.

Overall, both WHO estimates and national data confirm a slight decline in incidence since 2019. The notification rate decreased from 102.9 per 100,000 population in 2010 to 55.5 per 100,000 in 2024, alongside a parallel decline in estimated incidence. While incidence declined slightly, the detection gap widened from 30.5% in 2019 to 56.5% in 2024. At the same time, multidrug-resistant TB (MDR-TB) remains a significant concern, particularly among previously treated patients. According to the WHO Kyrgyz Republic TB country profile, MDR/RR-TB estimated, among 22% new cases and 49% of previously treated cases.^1^ Access to rapid molecular diagnostics and WHO-recommended shorter regimens remains essential.^7^

### Procurement and drug registration

Pharmaceutical registration and procurement of TB medicines in the Kyrgyz Republic have historically faced constraints related to the small market size and limited sales volumes, which discouraged some manufacturers from prioritising registration of new products. With support from the USAID Challenge TB project, several key medicines were registered, expanding the range of available formulations; however, some newer products, including bedaquiline, remained pending approval due to regulatory requirements. Although bedaquiline was not formally registered during the initial period of implementation, it was programmatically available through donor-supported mechanisms, including Global Fund–supported procurement channels.^8^ Following accession to the Eurasian Economic Union (EAEU), new regulatory procedures led to delays in re-registration of previously approved medicines, creating risks for uninterrupted availability. TB medicine procurement is organised through a mixed mechanism, with first-line and part of second-line drugs purchased through the State budget and the remaining share supplied via the Global Drug Facility, with United Nations Development Programme (UNDP) serving as principal recipient of Global Fund support.

Procurement specialists noted increasing complexity of customs clearance procedures, requiring multiple authorisations and extending delivery times. In response, the Ministry of Health, the National TB Center, and the Department of Medicines and Medical Devices have worked to strengthen procurement processes, including consideration of direct UN agency purchasing through the Global Drug Facility as a single source.^9^ With continued support from international partners, local pharmaceutical companies are also making efforts to sustain registration of essential TB medicines and ensure reliable patient access.^10^

### Case notification and access to care

Analysis of CLM data showed a persistent gap between the estimated number of people with TB and those formally notified in the Kyrgyz Republic. While WHO estimates suggest a slight decline in TB incidence since 2019, under-notification remained substantial and increased over time, from 30.5% in 2019 to 56.5% in 2024. The detection gap widened markedly in 2020, coinciding with COVID-19–related disruptions in access to diagnostic services, and remained high in subsequent years. As shown in the Figure, the detection gap increased sharply in 2020 and remained high through 2024. CLM highlighted several contributing factors. Patients in rural areas frequently reported financial and logistical barriers, including high transport costs and long travel distances to diagnostic centres. Stigma was also described as a major deterrent, delaying care-seeking and contributing to reduced notification. Health care providers noted that clinically diagnosed TB is included in the national registry; however, limitations in access to timely diagnostic services and challenges in obtaining bacteriological confirmation may partly explain why reporting gaps persist. Interviews with medical personnel further indicated that while large-scale interruptions in TB medicine supply have not occurred since 2019, human resource constraints remain significant. The declining number of trained TB specialists and limited entry of younger professionals place additional pressure on existing staff and may contribute to diagnostic delays, particularly in remote and underserved areas.

**Figure.**
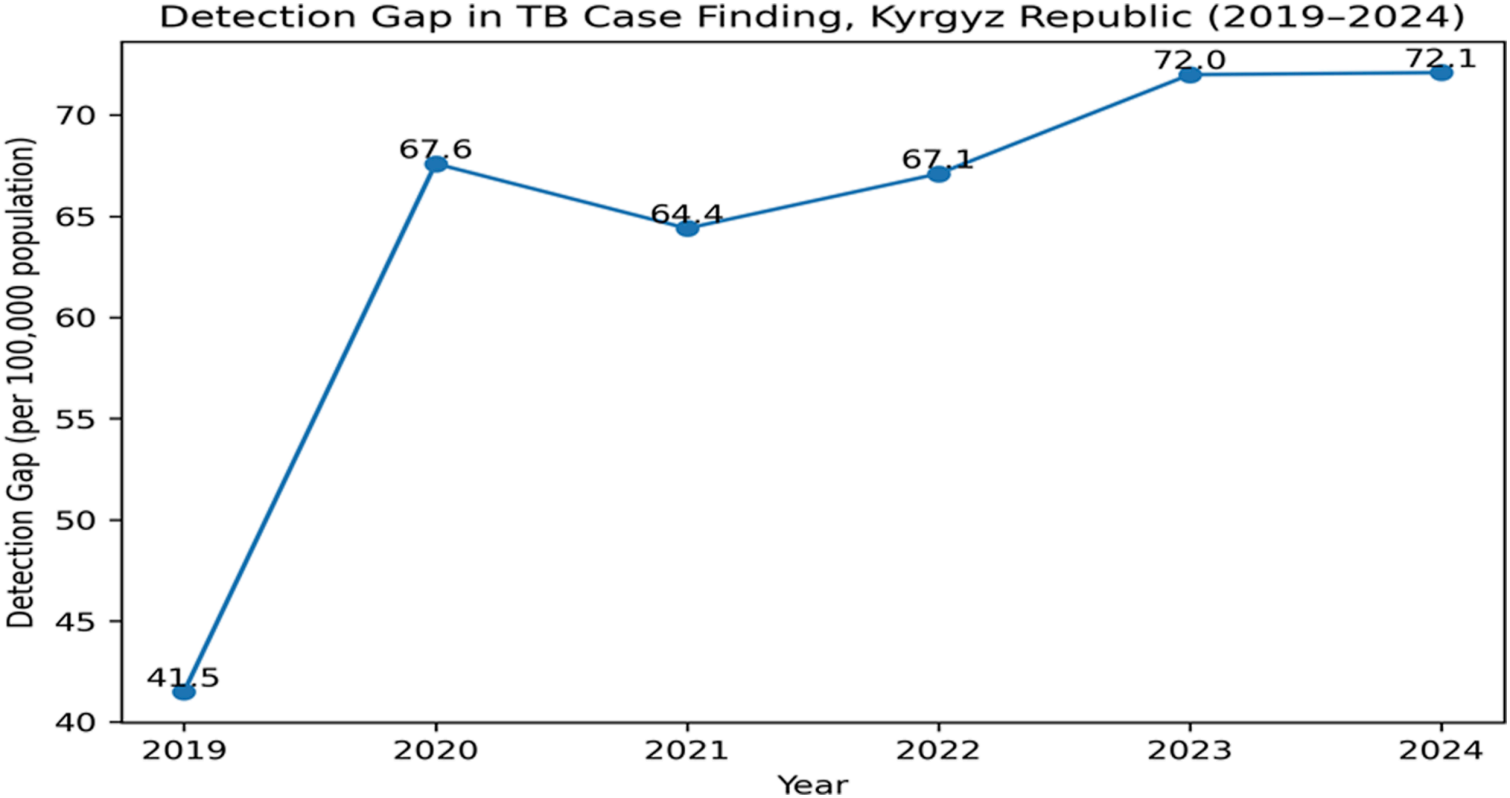
Detection gap between WHO-estimated TB incidence and notified cases in the Kyrgyz Republic, 2019–2024. The detection gap increased markedly in 2020 and remained high through 2024, reaching over 70 cases per 100,000 population, indicating persistent under-detection despite overall declines in TB incidence.

Overall, these findings indicate that strengthening case notification in the Kyrgyz Republic requires not only technical upgrades of surveillance systems but also expanded access to rapid molecular diagnostics, measures to address social barriers such as stigma and transport constraints, and sustained investment in human resources.

### Patient and community perspectives

CLM provided qualitative insights into the experiences of people with TB and frontline health workers in the Kyrgyz Republic. Patients frequently reported difficulties in accessing treatment, including delays and occasional shortages of medicines, which in some cases required continuation of older regimens with more pronounced side effects.^11^ Such interruptions were described as undermining trust in health services and negatively affecting treatment adherence. Some respondents described interruptions in treatment as affecting confidence in the health system. One patient noted that ‘treatment interruptions worsened my condition and reduced my trust in the health services’.

Awareness of new WHO-recommended TB medicines varied considerably. While some patients reported being informed and receiving newer regimens, others stated that they had not been offered these options. Health care workers acknowledged improvements in supply conditions in recent years but continued to highlight serious human resource constraints, with few young specialists entering the TB field. Misconceptions regarding the safety of new medicines were also reported, discouraging adherence and reinforcing stigma. As one respondent explained, ‘some patients believed that new medicines were being tested on them’, which discouraged adherence and reinforced stigma.

These findings underscore the importance of consistent patient education, reliable medicine supply, and sustained investment in human resources, and illustrate how CLM captures perspectives often overlooked in routine surveillance. Health care workers also highlighted staffing challenges. One provider noted that ‘there are fewer young specialists entering TB services, which increases workload and may delay diagnosis in remote areas’.

## DISCUSSION

This study represents the first systematic attempt in the Kyrgyz Republic to capture the perspectives of communities and health providers through CLM of TB services, conducted by TB People Kyrgyzstan in collaboration with KNCV KG. The findings point to encouraging progress as well as persisting challenges in ensuring timely diagnosis, uninterrupted treatment, and equitable access to essential medicines.

The barriers identified in the Kyrgyz Republic are consistent with findings from CLM initiatives in other high-burden settings. Similar challenges related to under-notification, disruptions in medicine supply, regulatory delays in introducing new drugs, stigma, and limited integration of TB services into primary health care have been reported across Eastern Europe, Central Asia, and other regions.

Despite an overall decline in TB incidence over the past two decades, under-notification remains a concern in the Kyrgyz Republic. Although people with clinically diagnosed TB are included in the national registry and initiated on treatment in accordance with national guidelines,^12^ limitations in access to diagnostic services and challenges in obtaining bacteriological confirmation contribute to persistent reporting gaps. Differences between WHO-estimated TB incidence and national notification data reflect methodological variations in estimation approaches and ongoing gaps in case detection. WHO estimates are derived from modelling that incorporates surveillance data and adjustments for under-reporting and under-diagnosis, whereas national figures represent only those individuals who are detected and formally notified within the health system. This gap underscores the need to strengthen active case finding, access to diagnostics, and reporting completeness.

The high proportion of drug-resistant TB among previously treated patients^13^ reinforces the importance of maintaining wide access to rapid molecular diagnostics and WHO-recommended shorter regimens.^14^ Progress in procurement and registration of TB medicines has been achieved; however, CLM findings indicate that some patients continued older regimens while awaiting access to newer drugs, and customs clearance procedures introduced following accession to the EAEU have prolonged delivery times. These findings highlight the need for continued efforts to streamline procurement processes and strengthen regulatory capacity.

A distinctive contribution of CLM lies in documenting how TB policies and service delivery are experienced at the level of people with TB and frontline health workers – perspectives that are not captured in routine surveillance systems. Respondents frequently cited stigma, transportation costs, and lack of psychosocial support as barriers that reduced care-seeking and treatment adherence. Some community members also expressed concerns about the safety of new medicines, reflecting a need for clearer communication, patient education, and trust-building measures. These findings highlight the need for counselling, awareness, and supportive services to improve engagement in care.

The findings point to several priority areas that require further attention:

1) Surveillance and notification: expand the use of rapid molecular diagnostics, strengthen active case finding, and ensure timely reporting of all people diagnosed with TB.
2) Procurement and supply chain: strengthen coordination among stakeholders, streamline regulatory and customs procedures, and minimise delays in the delivery of essential TB medicines.
3) Human resources: address shortages of TB specialists, particularly in rural and underserved areas, through targeted training, retention strategies, and incentives for young professionals.
4) Community engagement: institutionalise CLM within the National TB Program to sustain patient and community involvement and to strengthen accountability mechanisms.

The CLM initiative provided a perspective on TB service delivery in the Kyrgyz Republic. By combining programmatic data with community and patient voices, the study generated evidence that goes beyond routine surveillance and highlights the human dimension of TB care. Importantly, CLM demonstrated its potential not only as a tool for identifying service delivery gaps but also as a mechanism for building accountability, strengthening trust between communities and health authorities, and supporting health system resilience.

## CONCLUSION

Our study demonstrates that, despite progress in reducing TB burden and expanding access to new medicines, gaps remain in case notification, procurement processes, and patient-centred support. CLM effectively documented these challenges and amplified perspectives of people with TB and frontline health workers, including perceived delays in diagnosis, constraints in medicine availability, and persistent social and human resource barriers. CLM thus provided actionable evidence that complements national surveillance data and highlights the value of community engagement in strengthening accountability, communication, and trust between health authorities and affected populations. The findings were shared with the National TB Program and regional health authorities and informed discussions on aligning CLM insights with routine monitoring mechanisms and strengthening patient support systems. To accelerate progress towards the End TB targets, integration of CLM into routine programme monitoring should be considered, alongside sustained funding, expanded access to rapid molecular diagnostics, and strategies to address shortages of TB specialists. Embedding CLM within the National TB Program offers a pathway towards a more responsive, equitable, and resilient health system that is better prepared to address TB and future public health challenges.

## Supplementary Material
