# Supplementary material for: Community-led monitoring of TB services in the Kyrgyz Republic
Source: IJTLD Open. 2026 Jul 13;3(7):461–5. doi: 10.5588/ijtldopen.25.0691 (PMC13362295; doi:10.5588/ijtldopen.25.0691)
Supplement: Supplementary file 1 [file ijtldopen25-0691_supplementarydata1.pdf]

Supplementary File 1

Community-Led Monitoring Interview Guide

TB Services in the Kyrgyz Republic

Purpose

To collect qualitative information on barriers to TB diagnosis, treatment, and access to medicines from patients, health workers, and stakeholders.

Format

Semi-structured interviews (20–30 minutes)

Confidentiality

All responses are anonymous. Quotes may be used without identifying information.

Section 1. Respondent Information

Respondent type:

☐ Person with TB

☐ Health care worker

☐ Community monitor

☐ Other: _______

Region: __________

Sex: ☐ Male ☐ Female ☐ Other

Age group: ☐ <25 ☐ 25–34 ☐ 35–44 ☐ 45+

Interview date: __________

Section 2. Patients with TB

Access to diagnosis

How did you first access TB diagnostic services?

Probe: Was there any delay?

Did you experience difficulties reaching diagnostic services?

Probe: transport, distance, cost

Were diagnostic tests available at the first visit?

Probe: referral to another facility

How long did it take to receive diagnosis?

Were you informed about your results clearly?

Access to treatment

When did you start treatment after diagnosis?

Were all medicines available when treatment started?

Did you experience interruptions in treatment?

Were regimen changes explained to you?

Were you offered newer TB medicines?

Treatment experience

Did you experience side effects?

Did you receive support from health workers?

Did you receive psychosocial support?

Did transport costs affect treatment adherence?

Did stigma affect your treatment experience?

Recommendations

What challenges did you face during treatment?

What would improve TB services?

What additional support do patients need?

Section 3. Health Care Workers

What challenges do you face in TB service delivery?

Are TB medicines consistently available?

Are newer TB regimens accessible?

Are diagnostic tools sufficient?

Are staffing levels adequate?

Are there delays in treatment initiation?

What barriers affect case notification?

What challenges exist in rural areas?

How do medicine shortages affect patients?

What improvements are needed?

Section 4. Procurement and Policy Stakeholders

How is TB medicine procurement organized?

What are the main procurement challenges?

Are there delays in registration of new medicines?

What customs clearance barriers exist?

Are supply forecasting systems adequate?

How is coordination with NTP organized?

What causes medicine shortages?

How can community monitoring support procurement?

What improvements are needed?

Interview procedure

Semi-structured interview

Duration: 20–30 minutes

Audio recording with consent

Anonymous quotes may be used in analysis
